# Noninvasive temperature sensing technologies and the role of ferromagnetic nanoparticles in future applications

**DOI:** 10.1038/s41598-026-37266-8

**Published:** 2026-03-16

**Authors:** Antonia Ruffo, Matteo Busi, Markus Strobl, Michel Kenzelmann, Pierre Boillat

**Affiliations:** 1 Electrochemistry Laboratory, Paul Scherrer Institut, Forschungsstrasse 111, Villigen PSI, CH- 5232 Switzerland; 2 Laboratory for Neutron Scattering and Imaging, Paul Scherrer Institut, Forschungsstrasse 111, Villigen PSI, CH-5232 Switzerland

**Keywords:** Magnetic properties and materials, Nanoparticles, Imaging techniques

## Abstract

In polymer electrolyte fuel cells (PEFCs), temperature gradients can exert a substantial influence on cell performance and durability. Monitoring these gradients without perturbing fuel cell operation is one of the main challenges. This study introduces a novel method for remotely mapping fuel cell temperature using ferromagnetic nanoparticles, such as nickel and iron. These nanomediators possess temperature-dependent magnetic properties, enabling neutron depolarization imaging (NDI) to provide insights into the internal fuel cell temperature. We extensively evaluated the main parameters pertaining to the utilization of these nanoparticles in powdered form for temperature sensing. This encompassed an assessment of the minimum detection concentration and temperature sensitivity. Our findings reveal that while the smallest nanoparticles yield the highest relative change in depolarization, they exhibit considerably lower absolute depolarization coefficients. Hence, larger particles emerge as strong candidates for signal detection. Despite the challenges posed by the considerable size of these sensors, which inhibits in-situ dispersion, there is an opportunity to enhance nanoparticle characteristics. Such improvements could be achieved by scaling up the size of the materials from the nanoscale while retaining high magnetic saturation and temperature sensitivity.

## Introduction

Hydrogen technologies have emerged as particularly suited for long-term storage. In this context, the widespread adoption of fuel cell electric vehicles (FCEVs) is anticipated: this high-performance technology offers low-carbon transportation solutions^1–3^ and promises to be a suitable solution for heavy-duty and long-distance transportation.

Among the different types of fuel cells, polymer electrolyte fuel cells (PEFCs) are the most suitable for transportation applications because they offer advantages in terms of weight, volume, low operating temperature (80 °C), fast start, and the delivery of the highest power density^4^. A PEFC consists of a core membrane electrode assembly (MEA), including a proton-exchange membrane (PEM), catalyst layers, and a gas diffusion layer (GDL). In PEFCs, hydrogen gas is supplied to the anode, where it dissociates into protons and electrons at the catalyst surface. Protons are conducted through the PEM, which is typically made of a perfluorinated sulfonic acid polymer such as Nafion. The membrane selectively allows the passage of protons while being electrically insulating, forcing the electrons to flow in an external circuit, thereby generating electrical power. At the cathode, oxygen molecules are reduced by circuit electrons forming negatively charged hydroxyl anions, that react with protons to form H₂O.

In addition to the intended electrical output, a fuel cell produces heat and water as a result of its operation. These byproducts impact the life and performance of PEFCs. Since the temperature gradient in the core of the cell affects the evaporation and production of water, determining this gradient helps to understand flooding and dehydration in the different layers of the device, which can hinder its operation^5–7^. A lack of accurate water content management causes problems related to device durability, cost, and performance^5,8,9^. However, providing an efficient thermal detection method within the sealed MEA is a complex challenge.

One of the first approaches for measuring the membrane temperature was introduced by Mench et al. ^10^, in which micro-thermocouples were interposed between two Nafion membranes. However, this method was invasive due to the large dimensions of the sensors, which affects the fuel cell operation. He et al. ^11^ proposed the use of a gold foil, which improved the temperature sensitivity but hindered one-third of the proton transport through the membrane. Wang et al. ^12^ modified the interior of an MEA to conduct pyrometric measurements. The requirement for infrared transparency for pyrometric measurements complicated the system assembly and favored the formation of water between the bipolar plates, compromising detection accuracy. Lee et al. ^13^ introduced micro-electro-mechanical system (MEMS) sensors between two MEAs. However, the necessity to protect the sensor using a non-functionalized polymer such as parylene hindered the local transport of ions. Inman et al. ^14^ proposed one of the first non-invasive approaches by implementing phosphor particles as optical sensors on the surface of GDLs. The first limitation of this method arised from the need for extensive modification of the entire cell to ensure optical access. While the material was chemically and thermally resistant to the environment, the large size of the phosphor particles and their low-temperature coefficient limited the thermal detection accuracy.

Building on the idea of dispersing functional materials within the GDL, we propose a non-invasive thermal mapping method based on magnetic particles. By relying on temperature-dependent magnetic properties instead of optical emission, this approach avoids the need for optical access and allows the use of smaller particles, improving compatibility with the porous GDL structure and minimizing disruption to cell operation. During the development stage, the sensors are characterized across the temperature range from 30 to 100 °C with polarization neutron depolarization imaging (NDI). Unlike conventional neutron radiography, which has been extensively employed in PEFCs for mapping water distribution^15–18^, NDI has yet to be applied in these contexts. Extensive evidence from previous applications of polarized neutron imaging has established its effectiveness for detecting magnetic fields in ferromagnets^19,20^, with a sensitivity high enough to clearly distinguish these signals from the negligible magnetic fields (microtesla range) generated by operational fuel cell currents. Additionally, the technique has demonstrated sensitivity in identifying temperature-induced phase transitions near the Curie temperature, facilitating precise thermal mapping in critical areas^21,22^.

In this work, we explore the possibilities offered by micro- and nanoparticles of ferromagnetic materials for non-invasive temperature sensing applications, with the goal of using them for temperature mapping in the frame of *operando* studies of PEFCs. Using both in-house synthesized and commercially available materials, we combine experimental data with theoretical calculations to evaluate the impact of magnetic properties and particle size on material suitability for temperature sensing, using NDI as the characterization method. Considering the different and sometimes contradictory requirements of providing sufficient contrast, good temperature sensitivity, and particle size small enough to be integrated non-invasively in fuel cell porous media, we establish the basis for selecting these materials.

## Theoretical background

Neutrons, having no electric charge, exhibit extensive penetration through matter, thereby facilitating non-destructive neutron radiography. The neutron is a spin ½ particle and carries a magnetic moment **µ** (− 9.66 × 10^− 27^ J T^− 1^) aligned antiparallel to its spin. When a polarized neutron beam is coupled non-adiabatically to a magnetic field, the interaction between the magnetic moment of individual neutrons and the magnetic field can be described quasi-classically, whereby the neutrons undergo precession around the direction of the magnetic field^23,24^. The polarization vector (**P**) of such an ensemble of neutrons then behaves like a magnetic moment, **µ** ^25^, that is subjected to a torque, Γ, in a magnetic field **B** according to

1 $$\:\boldsymbol{\varGamma\:}=\boldsymbol{P}x\:\boldsymbol{B}$$

Consequently, the polarization vector rotates around the magnetic field axis, a phenomenon known as *Larmor precession*. Under these conditions, the neutron spin vector precesses around the magnetic field axis with an angular frequency equal to the Larmor frequency:

2 $$\:{\omega\:}_{L}=\gamma\:B$$

where γ is the gyromagnetic ratio (-1.76 ·10^11^ rad s^[- [1^ T^[- [1^).

Upon transmission through a ferromagnetic material, inhomogeneities in the magnetic induction within the material accordingly affect the neutron polarization vector accordingly. As it is possible to evaluate from the previous equations, the precession depends on the magnitude and orientation of the magnetic field (**B**), its orientation, and the neutron wavelength and path length—i.e., the duration the neutron spends in the field. Due to these considerations, polarization contrast neutron imaging studies can enhance the understanding of magnetic properties in ferromagnetic materials.

Halpern and Holstein^26^ deduced that, under the conditions where the magnetic domains possess a constant magnitude of the magnetic field with random and isotropic orientation, the resulting neutron depolarization after transmission can be described as:

3 $$\:\frac{P}{{P}_{0}}={e}^{\frac{-{\gamma\:}^{2}{B}^{2}d\delta\:}{3{v}^{2}}}$$

where v is the neutron velocity, B is the magnetic field of each individual domain, d is the sample thickness, and δ is the domain size. For the current study, however, the analyzed samples were prepared as powder mixtures to investigate the impact of magnetic powder concentration and simultaneously simulate the environment in an ex-situ manner for a gas diffusion layer dispersion. Consequently, it is essential to apply a correction to the equation, converting the sample thickness (d) into the effective thickness of the transmitted magnetic material phase. For this the sample porosity and the ferromagnetic volume fractions of the two mixed materials must be considered, yielding the effective thickness d_eff_:

4 $$\:{d}_{eff}={\varphi\:}_{s}\cdot(1-\varepsilon\:)\cdot\:d$$,

5 $$\:{\varphi\:}_{s}=\:\frac{\raisebox{1ex}{${m}_{s}$}\!\left/\:\!\raisebox{-1ex}{${\rho\:}_{s}$}\right.}{\raisebox{1ex}{${m}_{s}$}\!\left/\:\!\raisebox{-1ex}{${\rho\:}_{s}$}\right.+\raisebox{1ex}{${m}_{PTFE}$}\!\left/\:\!\raisebox{-1ex}{${\rho\:}_{PTFE}$}\right.}$$.

Here φ_s_ is the volume fraction of magnetic sample material relative to the solid material phases, m_S_ and m_PTFE_ are the masses of the magnetic sample material and PTFE, respectively, ρ represents the respective material densities, ε is the porosity (considered equal to 50%) of the magnetic powder/PTFE mixture, and d is the total sample thickness.

A further consideration concerns the assumed magnetic field B of each individual domain. In the domain theory, Weiss postulates that each domain is spontaneously magnetized to the saturation value (M_s_)^27^. Thus, here, we treat the measured saturation magnetizations as representative of the domain magnetic field of the ferromagnetic powders used in the study. Additionally, for the following calculations, the assumption that crystallite size equals domain size is adopted. Knowing the magnetic field, domain size, and effective thickness of the samples allows us to predict the outcome of the depolarization measurements which can be compared to the actual results of our measurements.

### Experimental section

#### Material selection

**Fig. 1:**
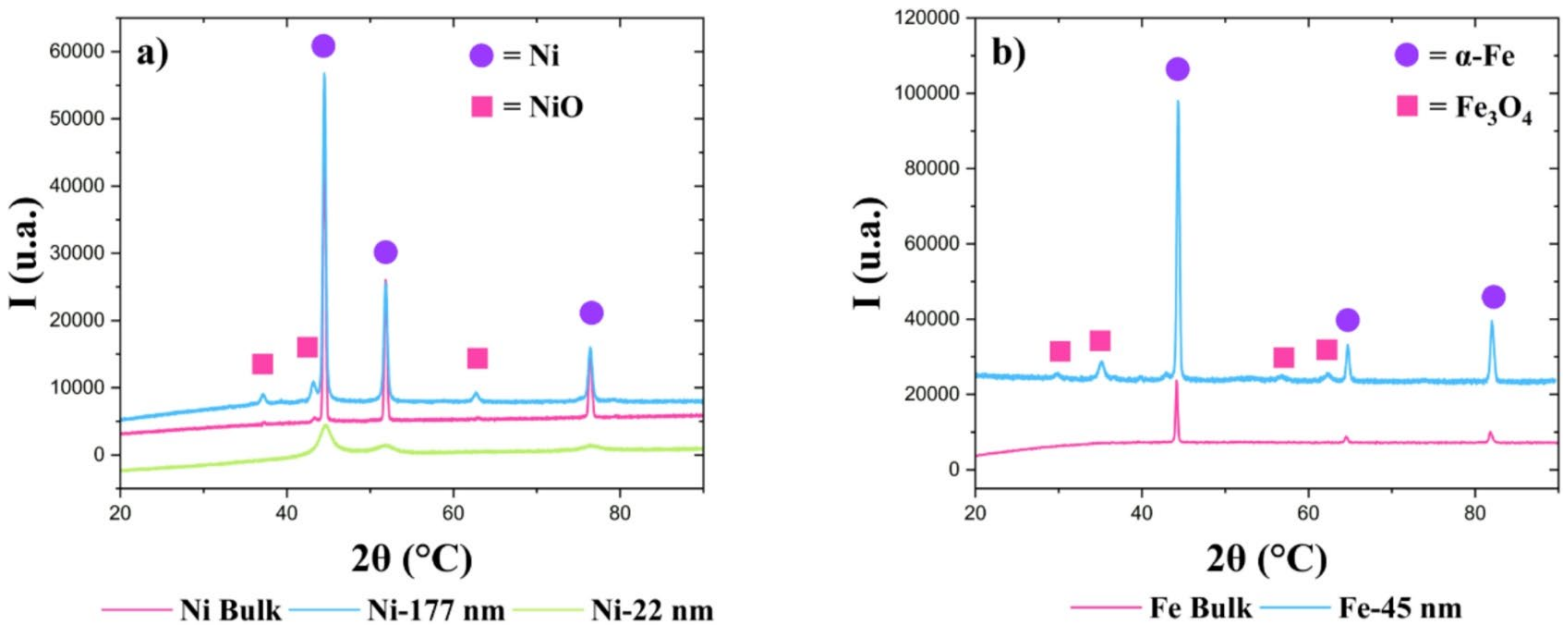
Powder X-Ray Diffraction analysis of nickel (a) and iron (b) samples. The results show that these materials present the expected crystallographic phase. However, in some samples, it is possible to notice a passivation, given the presence of the oxides of the corresponding metals.

Due to their ferromagnetic properties, nickel and iron powders were chosen for this study^28,29^. Among their ferromagnetic characteristics, two key properties are particularly relevant: high magnetic saturation^30^, which leads to enhanced detectability in polarization contrast neutron imaging measurements, and a low Curie temperature^31,32^, enabling high-temperature sensitivity. In this study, we examined nickel and iron powders with different grain sizes to investigate their properties: nickel (99.8%) and iron (99.0%) bulk powders were acquired from Goodfellow Cambridge Ltd. (Huntingdon, England). Nickel nanoparticles (≥ 99% ) (177 nm) with a specified size of 100 nm and iron nanoparticles (99.5%) (45 nm) with a specified size of 25 nm were acquired from Sigma-Aldrich Chemie GmbH (Taufkirchen, Germany). Smaller nickel nanoparticles (22 nm) were synthesized in-house as described in the “Nickel-22nm: polyol synthesis” paragraph. All samples were characterized by X-ray diffraction (XRD), as shown in Fig. 1.

**Fig. 2:**
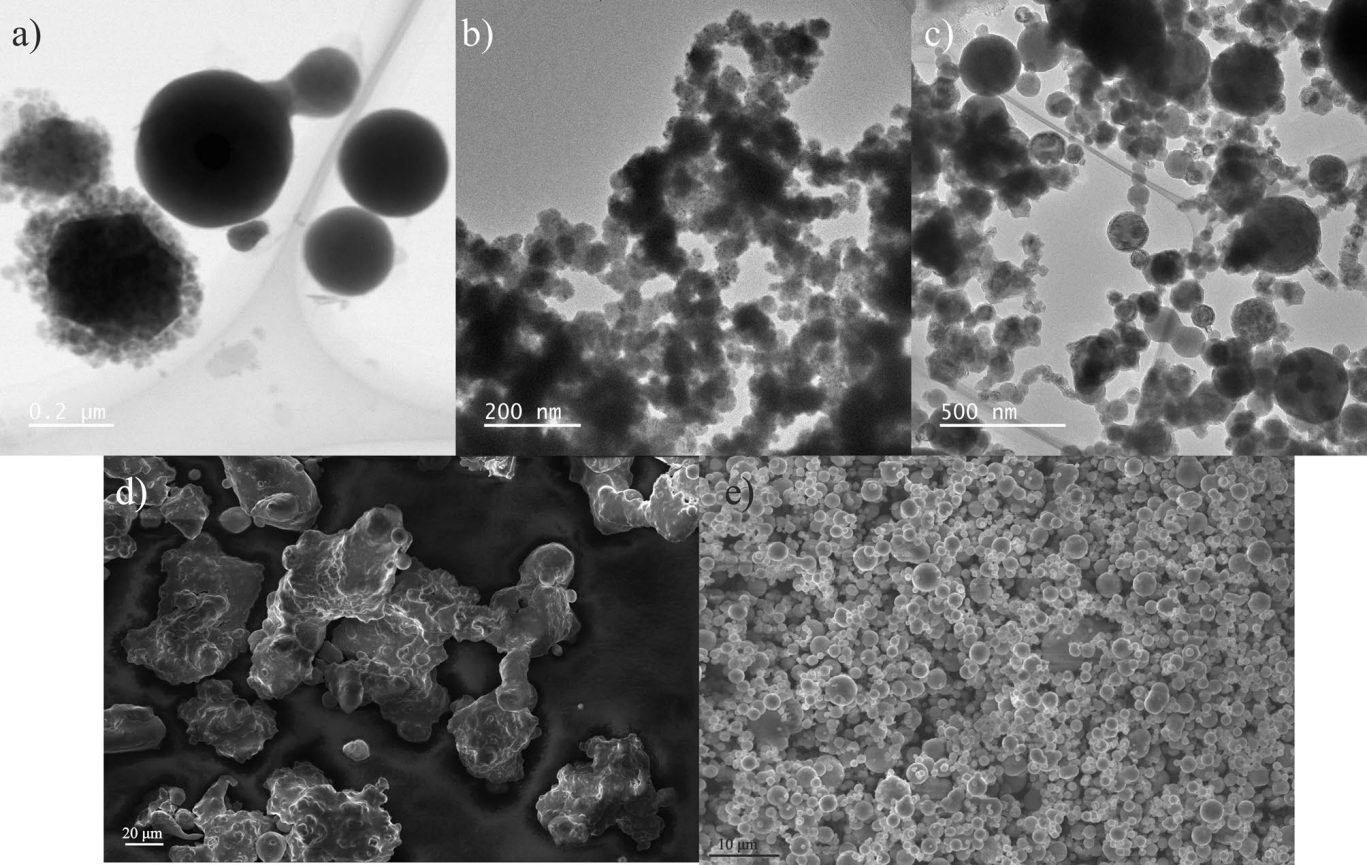
TEM of (a) Ni-177 nm, (b) Ni-22 nm, (c) Fe-45 nm, and SEM of (d) Ni bulk, and (e) Fe bulk.

In addition to these structural characterizations, transmission electron microscopy (TEM) for the nanosized samples and scanning electron microscopy (SEM) for the bulk samples were conducted to determine the particle size values (Fig. 2). The particle dimensions are measured individually from the TEM images, and the mean particle size is determined by averaging these measurements. Despite particle aggregation, a spheroidal and slightly faceted grain morphology is observed in all materials except for Ni bulk, which exhibits an irregular shape. Crystallite size and impurity phases were evaluated by Rietveld refinement^33^, whose results are listed along with the particle sizes in Table 1. The goodness-of-fit was evaluated using the weighted profile factor (R_wp_), which acceptable value results in values below the threshold 10% for typical X-ray data, according to the guidelines^33^.

**Table 1:** Summary of the selected samples of nickel and iron powder and their corresponding particle size, crystallite size, impurities, and the weighted profile values (R_wp_).

Sample	Particle size (nm)	Crystallite size (nm)	Impurity (%)	Impurity type	*R*_wp_ (%)
*Ni bulk*	100·10^3^ ± 47.3	500	4.5	NiO	3.40
*Ni-177 nm*	138 ± 11.46	177	26.3	NiO	6.51
*Ni-22 nm*	44 ± 2.24	22	-	-	6.51
*Fe bulk*	2378 ± 14.22	117	-	-	3.47
*Fe-45 nm*	139 ± 12.53	45	0.6	Fe_3_O_4_	1.51

## Nickel-22 nm: polyol synthesis

The nickel nanoparticles denoted ‘Ni-22nm’ were synthesized in-house through the polyol method^34^, a solution synthesis used for a higher tunability of the size and shape of nanomaterials. However, in the following experiment, the synthesis was modified by omitting the capping agent (Polyvinylpyrrolidone, PVP). This was done in order to obtain particles within the nanometric size range while maintaining suitable magnetic properties for sensor applications. The redox reaction is described by the following chemical equilibrium:

$$\:2{\mathbf{N}\mathbf{i}}^{2+}\left(\mathbf{e}\mathbf{g}\right)+\mathbf{B}{\mathbf{H}}_{4}^{-}\left(\mathbf{s}\right)+2{\mathbf{H}}_{2}\mathbf{O}\:\left(\mathbf{e}\mathbf{g}\right)\to\:2\mathbf{N}\mathbf{i}\:\left(\mathbf{e}\mathbf{g}\right)+2{\mathbf{H}}_{2}\left(\mathbf{g}\right)+4{\mathbf{H}}^{+}\left(\mathbf{e}\mathbf{g}\right)+\mathbf{B}{\mathbf{O}}_{3}^{-}\left(\mathbf{e}\mathbf{g}\right)$$

0,025 mmol of Ni(acet)·4(H_2_O) (99.995%, Merck, Darmstadt, Germany) were dispersed in 250 mL of ethylene glycol (EG) (≥ 99%, Merck, Darmstadt, Germany) inside a 250 mL flask. After attaching a condenser to the flask, the solution was stirred with a magnetic anchor at 373 K for 2 h. 200 mg of NaBH_4_ (≥ 99%, Merck, Darmstadt, Germany) were gently introduced in the green solution, causing the color to change from green to black. The resulting colloidal dispersion was washed with 400 mL of ethanol, centrifuged, and air-dried at room temperature.

## Sample Preparation

The commercial and in-house nickel and iron powder samples were prepared by mixing the powders with polytetrafluoroethylene powders (PTFE, 500 μm, Goodfellow Cambridge Ltd., Huntingdon, England) to mimic the actual environment inside a fuel cell. Samples with nanoparticle concentrations equal to 1%, 10%, and 50% weight on weight (w/w) were prepared through a solid mixing method. The mixtures were stirred using a plastic or glass rod until a homogeneous color and graininess were achieved. The different mixtures were used to identify the minimum detection concentration.

For the study of the dispersion of the following sensors inside a porous medium such as GDLs, it is convenient to evaluate the amount of material analyzed in terms of volume. For this reason, the following tables show the density values of the powders, the volumes of the samples at the different concentrations, and their integrated thickness.

## Magnetic characterization

Magnetic properties of the powders were evaluated using a SQUID magnetometer (Quantum Design MPMS-5 S). Each prepared mixture was wrapped in Teflon tape, placed in a brass holder and then aligned in the instrument chamber. The magnetization hysteresis loops were measured by applying magnetic fields in the range of ± 10k Oe, over the temperature range of 300–400 K in increments of 10 K.

### Neutron experiment set-up

**Fig. 3:**
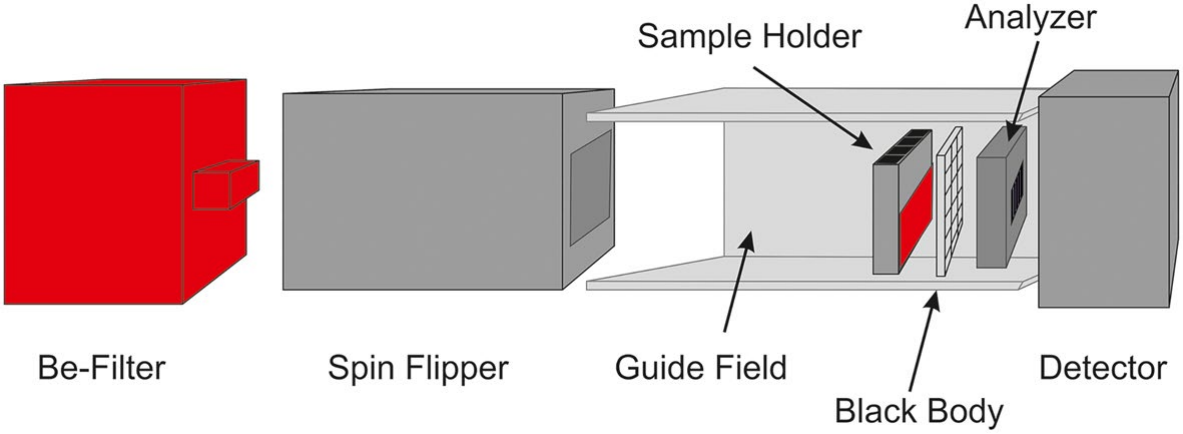
Selected depolarization neutron imaging setup.

The measurements were performed at the SINQ neutron source of the Paul Scherrer Institute utilizing the polarized neutron instrument BOA (Beamline for neutron Optics and other Approaches)^35^. BOA provides a neutron spectrum with a wavelength range of 1–20 Å, with a spectral peak at λ = 2.8 Å and a mean wavelength of λ = 3.8 Å. A multichannel polarizing-bender unit is installed in the BOA beam extraction section within the shielding monolith of the neutron source. The bender consists of bent channels of FeCo/TiZr supermirrors on anti-reflective TiZrCd substrates and acts as a neutron spin filter, transmitting only one spin state^36^. The average instrumental white-beam polarization was measured at the beam exit from the monolith where a pinhole is placed to be approximately 96.6%^37^. For this experiment, the set-up illustrated in Fig. 3 was installed. During measurements, the spectrum below 4 Å was suppressed using a beryllium filter. Neutrons with longer wavelengths exhibit heightened sensitivity to magnetic fields, attributable to their reduced velocity, extending their interaction time and increasing detection efficiency.

In the polarization contrast neutron imaging setup, a remote-controlled spin flipper was used to invert the spin orientation through an adiabatic resonance^38^, allowing switching between spin-up and spin-down polarized incident beams. The neutron polarization orientation was maintained throughout the beamline using guide fields, a piece of equipment consisting of permanent magnets mounted on soft iron plates that produce a weak homogeneous field (5.5 mT) oriented parallel to the spin-up polarization at the sample position. The sample holder was positioned inside the guide field and consisted of an aluminum block with 16 holes, each capable of accommodating a cuvette. Thermal films were affixed to the walls to ensure uniform heating and temperature regulation within the sample environment. Downstream of the sample position the polarization analyzer—a supermirror-based neutron spin filter (NSF) transmitting only the spin-up state, similar to the polarizer—was mounted. This device limited the field of view to 4 × 4 cm^2^ and was positioned in front of the detector. In close contact with the analyzer black-body (BB) assembly was installed. The BB assembly consists of an aluminum grid with regularly spaced dots of neutron absorbers (boron). The BB is used to apply a background correction for scattered neutrons^39^. At the end of the beamline is positioned the detector, which consists of a light-tight box containing a scintillator screen (Gd_2_O_2_S: Tb pure; 20 μm) facing the beam, which converts absorbed neutrons into visible light. The emitted light was recorded by a CCD camera (Andor Ikon-M, 1024 × 1024 pixels, sensor cooled down to -70 °C) equipped with a Zeiss Makroplanar Milvus 100 mm f/2 M objective lens.

Using the described setup, neutron images were acquired for both spin-up and spin-down polarized incident beams. For each polarization setting, 15 images were recorded, with each image having an exposure time of 2 min, resulting in a total acquisition time of 30 min per spin state—1 h per field of view. This procedure was repeated at eight temperatures ranging from 30 to 100 °C. After reaching the target temperature, images were acquired following a stabilization period: 15 min for temperatures between 30 and 70 °C, 30 min at 90 °C, and 90 min at 100 °C, ensuring thorough thermal stabilization.

## Imaging processing

The acquired images were processed to reduce stochastic errors, such as white spots and shot noise, as well systematic errors, such as the scattered background and other biases. White spots are caused by gamma and X-rays stemming from the neutron source or from the neutron captures in the scintillator screen, which directly hit the CCD. To address this issue, an outlier filter was applied, replacing pixel values with the median value of their neighboring pixels whenever their difference exceeded a predefined threshold. A three-dimensional neighborhood of 3 × 3 × 3 pixels was used including all three images recorded for the same state in the analysis. Limited counting statistics of both the neutrons captured by the scintillator and photons reaching the CCD cause shot noise, which was corrected by applying a Gaussian filter, that reduces noise at the expense of image resolution. An additional source of systematic error is the background caused by neutron scattering, which can be corrected using the black body (BB) grid. The absorbing dots of the BB grid prevent neutron transmission, implying that intensity detected at the locations of the dots corresponds to background scattering. These background values measured at the dots locations can be interpolated to the full image background to be subtracted from the image. Standard protocols are available for this correction^39^.

Based on the neutron imaging measurements obtained using spin-up (I_+_ (x, y)) and spin-down polarized beam (I_-_ (x, y)), the neutron polarization can be calculated as follow:

6 $$\:P\left(x,y\right)=\:\frac{{I}_{+}\:\left(x,y\right)-{I}_{-}(x,y)}{{I}_{+}(x,y)+{I}_{-}(x,y)}$$

The equation is applied pixel-wise to obtain the local polarization of the beam after transmitting the samples, thus imaging the depolarization induced by the sample. These polarization images are finally normalized by the open beam polarization image (P_0_(x, y)), i.e., the image captured with all setup elements in place except the sample material, to yield the relative polarization image (P_rel_)

7 $$\:{P}_{rel}\left(x,y\right)=\:\frac{P(x,y)}{{P}_{0}\:(x,y)}$$

Regions of interest (ROI) corresponding to the different samples were selected from these images to evaluate the neutron beam depolarization caused by each sample.

## Data processing

In order to assess the sensing characteristics of the selected samples, additional data processing was performed. The depolarization coefficient (η) ^40^ describes the ability of a specific material to depolarize the neutron beam, analogous to the attenuation coefficient in the Beer-Lambert law, as:

8 $$\:\eta\:=\:\frac{-ln\left({P}_{rel}\right)}{{d}_{eff}}$$

where d_eff_ is the effective neutron path length calculated according to Eq. (4).

Temperature sensitivity in ferromagnetic powders refers to the quantitative measurement of how their magnetic properties vary with temperature. As relevant to their potential use as temperature-sensing material, in the neutron polarization data, this feature is determined by the *relative depolarization variation*:

9 $$\:{k}_{T}=\frac{\eta\:\left(T\right)}{\eta\:(30\:^\circ\:C)}$$

defined as the depolarization coefficient at temperature T normalized by the depolarization coefficient to its value at T = 30 °C.

The absolute depolarization variation Δη(T) is the key parameter for the temperature-sensing application. It describes the absolute change of the depolarization coefficient at temperature T relative to η(30 °C):

10 $$\:\varDelta\:{\upeta\:}\left(T\right)=\:{\upeta\:}\left(T\right)-\:{\upeta\:}(30\:^\circ\:C)$$

These last two parameters are crucial role in characterizing magnetic materials for temperature-sensing applications. However, the absolute depolarization variation is particularly significant due to its unique capacity to encompass both thermal sensitivity and responsiveness to neutron interactions. This dual capability is especially important when deploying sensors in enclosed environments, where enhancing detection capabilities can pose significant challenges. Therefore, this last parameter assumes heightened importance in comprehending the application of these sensors for their intended purpose.

## Results and discussion

### Magnetic properties

Magnetic characteristics such as magnetic saturation (M_s_), magnetic remanence (M_r_), and coercive field (H_c_) were determined from the magnetization hysteresis.

As previously explained in the description of the Halpern and Holstein equation (Eq. 3), it is essential to consider the magnetic field associated with each domain, as it significantly influences the precession of the neutron polarization vector. Given that the magnitude of this field corresponds to the saturation magnetization, the latter parameter primarily contributes to image contrast and, consequently, the depolarization signal.

Remanence, defined as the ability of a material to retain its magnetization after exposure to an external magnetic field, significantly influences neutron depolarization. Specifically, materials in a remanent state can induce varying degrees of neutron depolarization due to differences in the orientation of their magnetization vectors. When remanence maintains an orientation perpendicular to the neutron polarization vector, neutron precession is effectively enhanced, resulting in increased depolarization levels.

Coercivity plays a crucial role determining the orientation stability of magnetic domains. This parameter is formally defined as the magnetic field strength required to completely demagnetize a material. In the context of depolarization neutron imaging, materials characterized by a high coercivity, which exceeds the strength of external magnetic fields, such as those generated by guide fields within the experimental setup, hold relevant importance. This inherent characteristic prevents unintended changes in magnetic properties induced by the guide field, thus guaranteeing the reliability of the experimental results. By minimizing undesired magnetic effects during the imaging process, these materials significantly contribute to the precision and reliability of neutron imaging procedures.

**Fig. 4:**
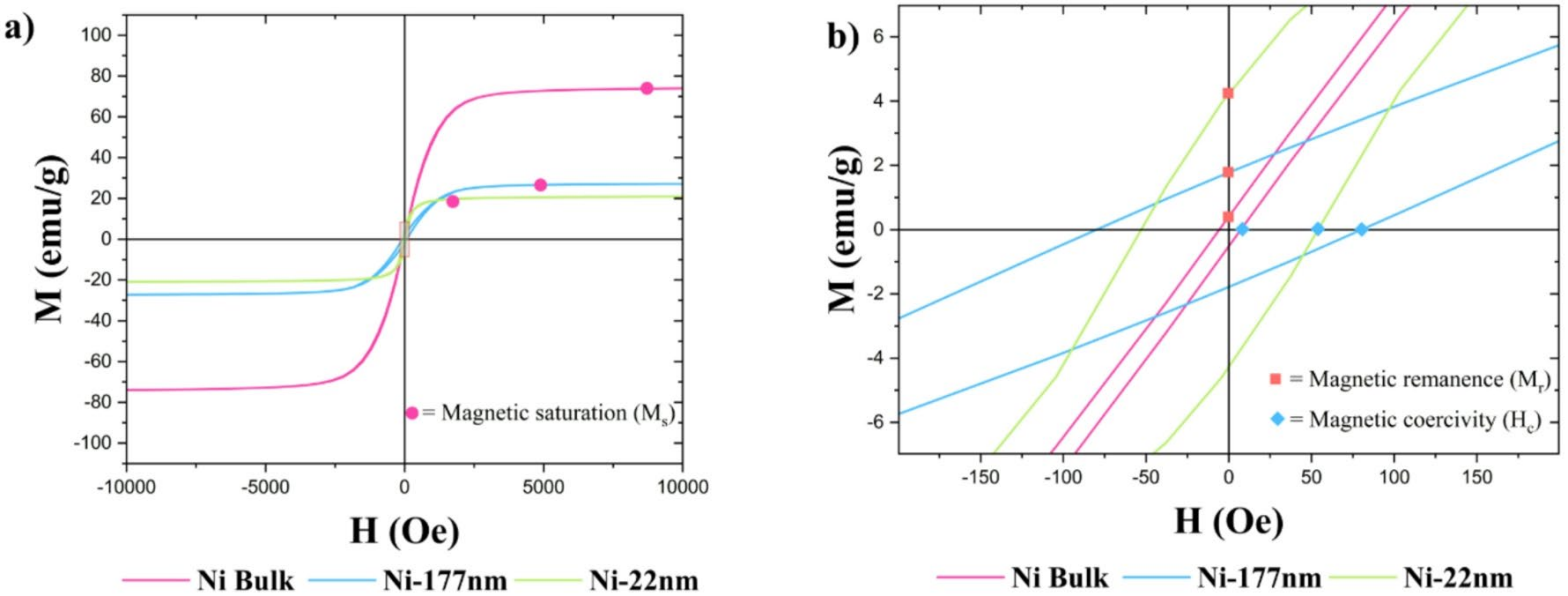
(a) Nickel magnetization hysteresis at 300 K and (b) an enlarged representation of the central details revealing remanence and coercive field.

**Fig. 5:**
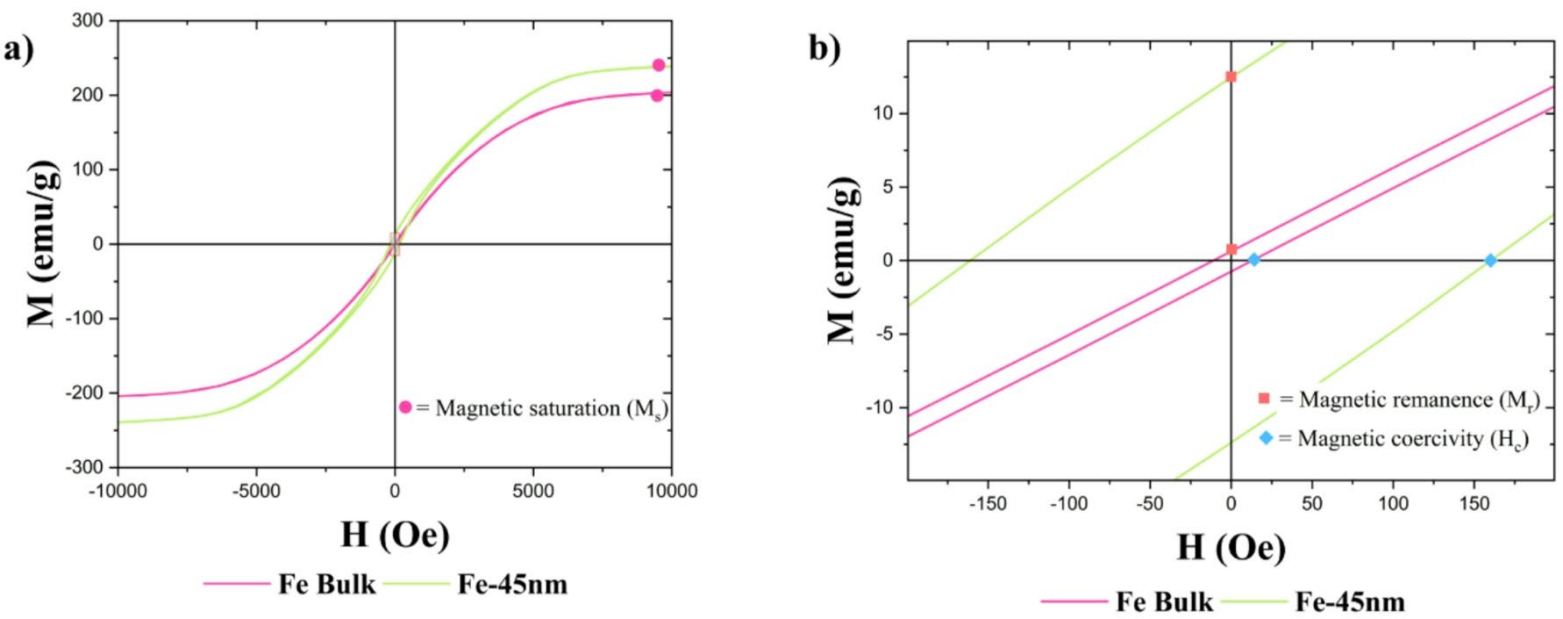
Iron magnetization hysteresis at 300 K and an enlarged representation of the central details revealing remanence and coercive field.

The above discussion analyzes these magnetic parameters primarily to characterize the various candidate sensors. Notably, the following study exclusively concentrates on using unmagnetized materials in a field of 5.5 mT, a condition where remanence and coercivity have minimal relevance in the NDI analyses. Here, the data of all nickel and iron samples at temperatures equal to 300 K are reported (Figs. 4 and 5). A variation of the magnetic behaviour depending on the grain size is observed.

The relevant parameters are collected in Table 2.

**Table 2:** Crystallite size and collection of magnetic saturation, magnetic remanence and coercivity field at 300 K.

Material	Crystallite size (nm)	Saturation (emu/g)	Remanence (emu/g)	Coercivity (Oe)
*Ni bulk*	500	74.02 ± 26.96	0.81 ± 0.33	5.5
*Ni-177 nm*	177	27.19 ± 14.25	1.69 ± 0.06	81.0
*Ni-22 nm*	22	20.92 ± 9.38	3.95 ± 0.62	55.0
*Fe bulk*	117	202.30 ± 8.16	0.38 ± 0.33	10.0
*Fe-45 nm*	45	238.00 ± 9.81	12.17 ± 1.70	161.0

For decreasing Ni particle size, the main observed effect is that the magnetic saturation decreases. By maximizing the area-to-volume ratio, a higher fraction of surface atoms is exposed, leading to a higher incidence of dangling bonds, which can act as defects. Consequently, a different electronic configuration of these atoms is verified, leading to a lower contribution to the overall particles magnetic moment^41^. However, it is important to note that XRD results reveal the presence of NiO impurities in both Ni bulk and Ni-177 nm samples. Although these impurities constitute only 4.5% in the Ni bulk and about 26.3% in the Ni-177 nm, they can significantly reduce the magnetic saturation compared to pure Ni nanoparticles of similar dimensions^42^. An opposite trend is observed for the remanence. A possible explanation is that the nanoparticles exhibit a transitional behavior between single (SSD) and multi-domain (MD) states^43,44^. Biedermann and Parés^43^ show that, for several studies, the coercivity fields increase across particle size in the SSD range, reaching a maximal peak on the border to the SSD-MD transition. In the nickel magnetization data presented in Table 3, a maximum value for the coercivity is observed in the intermediate nickel sample. From these considerations, it is possible to assume that our Ni-22 nm particles are in a single domain state.

**Table 3:** Density of the powder included in the study.

Material	Density (g/cm^3^)
*PTFE*	2.00
*Nickel*	8.90
*Iron*	7.87

In the case of the iron samples, it is noted that the magnetic saturation increases with decreasing size, contrary to the outlined expectation. This can be explained by the presence of iron oxide impurity, whose magnetic field may interact with the primary iron phase, enhancing the total magnetic saturation of the material^45^.

### Polarization contrast neutron imaging

**Fig. 6:**
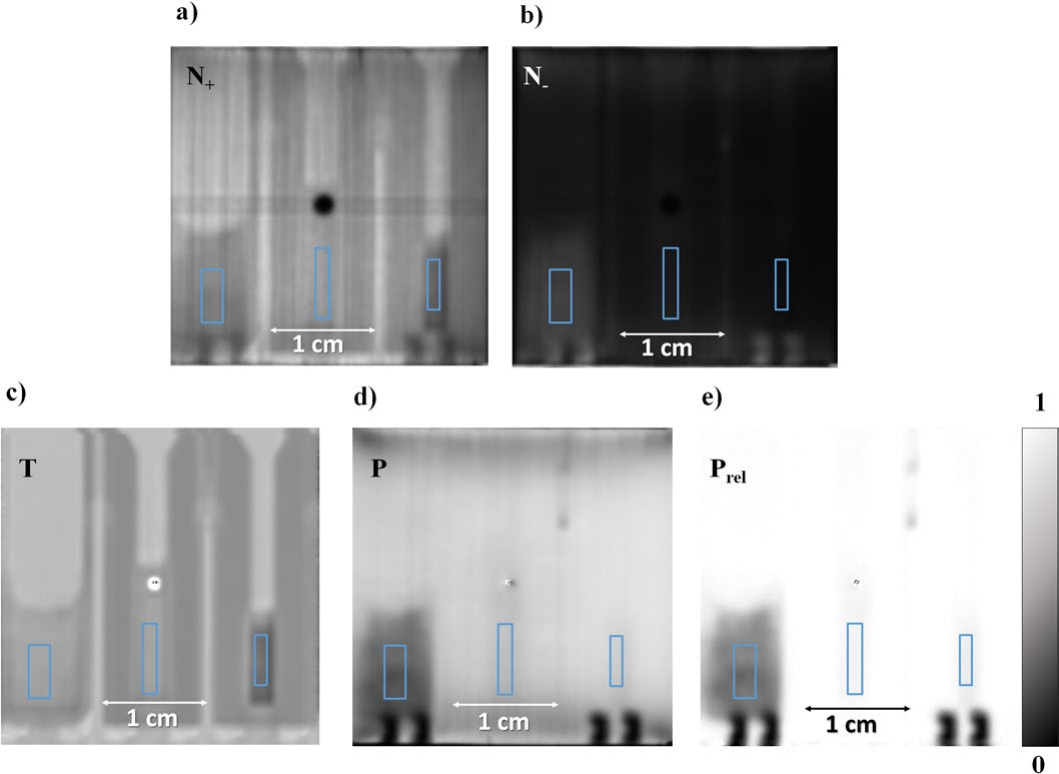
Imaging illustrations at 30 °C of nickel samples (from left to right, Ni bulk, Ni-177 nm and Ni-22 nm) in the condition of: (a) Spin up, (b) Spin down, (c) Transmission, (d) Polarization, and (e) Relative polarization.

Figure 6 shows the images of the nickel samples at 30 °C, where (a) and (b) represent the spin-up and spin-down images, respectively. In these images, apart from exhibiting distinguishable differences among the images corresponding to different spin states, it becomes evident that the samples also exhibit contrast variations. Specifically, in the spin-up image, the analyzer filters out the neutrons whose spins have deviated from their original orientation, resulting in an image that exhibits contrast while preserving the transmission information - visible in image c). Conversely, the spin-down image captures the neutron spins that vary its direction towards an upward orientation upon interacting with the magnetic fields. This results in a discernible change grayscale intensity, particularly in regions containing magnetic material. As described in the Imaging processing section, the images were processed by applying Eqs. 6 and 7 pixel-wise in order to obtain the polarization image (d) and the relative polarization image (e). The magnetic powders, marked by the blue rectangles (defined as ROIs), contribute differently to beam depolarization, thereby influencing the contrast exhibited on the grayscale. The grayscale in these two images reflects the polarization values ranging from 0 (indicating complete beam depolarization) to 1 (indicating total beam polarization). This enables the assessment of the magnetic interactions within the sample materials.

In order to understand and identify useful materials for temperature sensing applications, the average of each ROI is analyzed as a function of concentration, grain size, and type of material.

### Impact of concentration

**Fig. 7:**
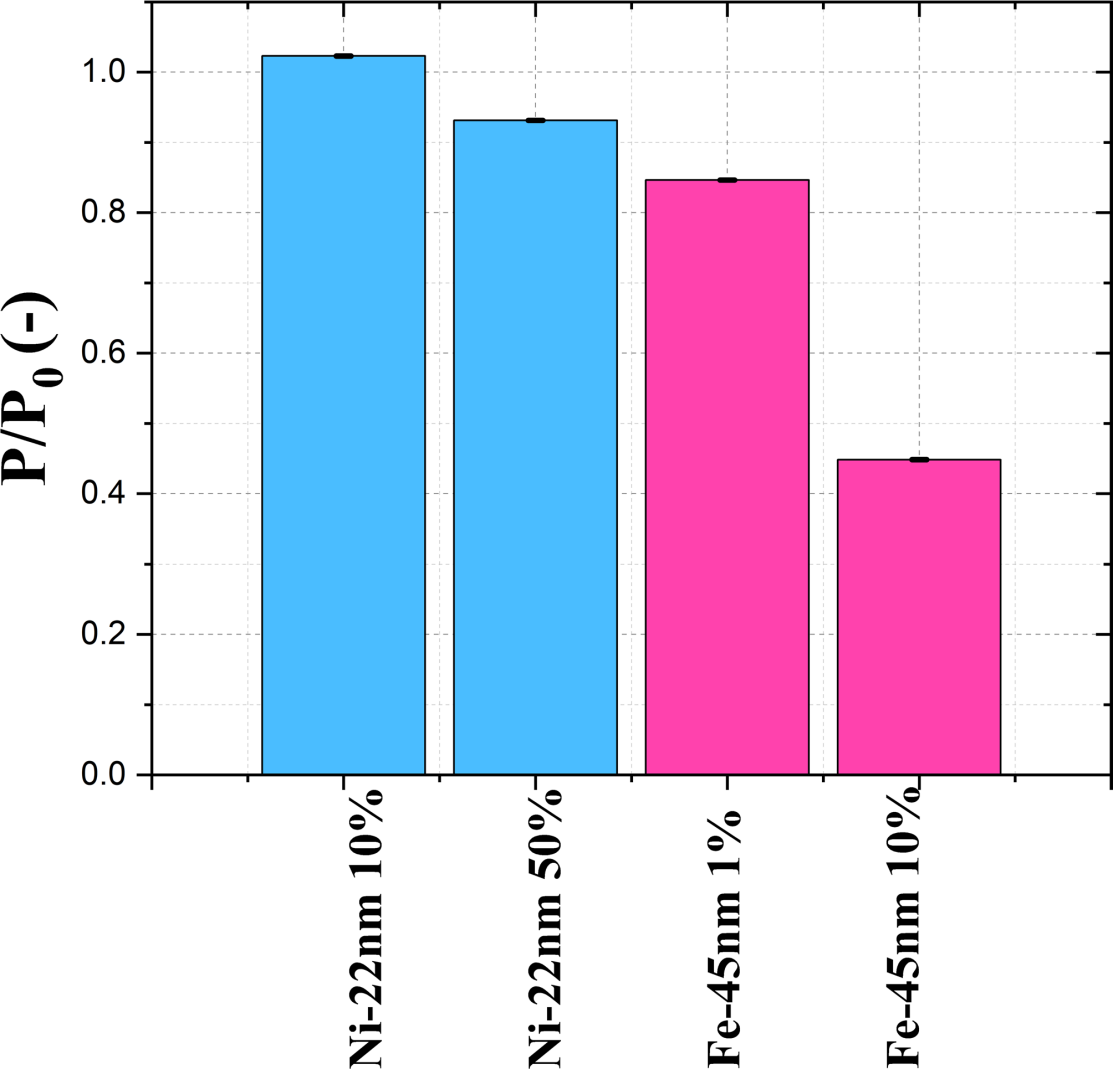
Neutron polarization of Ni-22 nm (blue) concentrated 10% and 50% w/w in PTFE powder and Fe-45 nm (pink), concentrated 1% and 10% w/w in PTFE powder performed at 30 °C.

Detection sensitivity is a fundamental characteristic in the field of magnetic sensing, which can be assessed through investigations of magnetic particles at various concentrations. In Fig. 7 polarization data of iron and nickel nanoparticles mixed with PTFE powders at concentrations of 1%, 10%, and 50% w/w with PTFE powders are reported. As expected from Eqs. 3–5, a higher concentration leads to a higher depolarization, due to the increased effective thickness of magnetic material along the neutron path. Even at very low concentrations (1%), the iron samples show a measurable depolarization signal. However, for Ni-22 nm nanoparticles, a concentration of 10% w/w is insufficient for a reliable detection.

From these considerations, following the calculations in Table 4, we conclude that Ni-22 nm cannot be reliably detected for a volume fraction equal to 2.44%, requiring a minimum volume fraction of approximately 7.5 times higher. Due to this restriction, Ni-22 nm was studied only at 50% w/w for the remainder of this study. On the other hand, iron nanoparticles can be detected even for lower volume fractions compared to nickel nanoparticles, allowing the use of a smaller amounts of material. The obvious advantage of using the lowest possible volume fraction is the requirement to be as non-invasive as possible. For this reason, in terms of concentration, Fe-45 nm samples are the most promising for the use in real fuel.

**Table 4:** Volume and integrated thickness of nickel and iron powders at different concentration.

Sample	Integrated thickness (cm)	Volume fraction (%)
*Nickel 10% w/w*	0.01218	2.44
*Nickel 50% w/w*	0.09174	18.35
*Iron 1% w/w*	0.00128	0.26
*Iron 10% w/w*	0.01373	2.75

### Impact of size and material

**Fig. 8:**
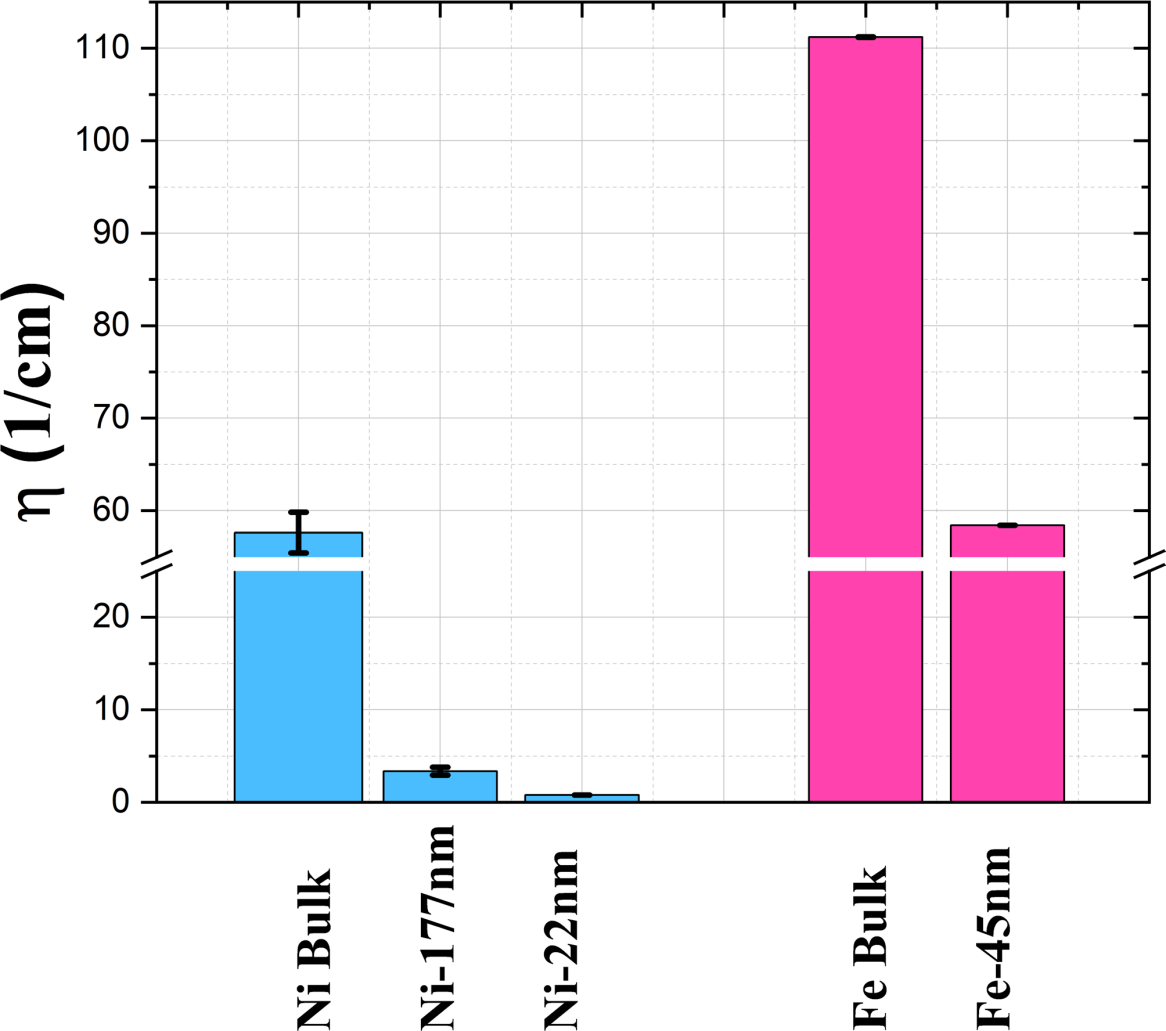
Depolarization coefficient of nickel (blue) and iron (pink) samples. All coefficients are measured with a weight on weight (w/w) concentration of 10%, except for the Ni-22 nm sample where a concentration of 50% w/w is used.

An additional requirement for nano-sensing applications is understanding how neutron polarization varies as a function of the grain size. By comparing materials at the same concentrations, a higher neutron depolarization is observed for the bulk powders (Fig. 8). This observation is consistent with Eq. (3), from which it is expected that materials with larger magnetic domains and higher magnetic saturation yield a higher depolarization.

Figure 8 shows the measured depolarization coefficient of the different powders. The values appear to be higher for the iron samples, as they possess a stronger magnetic fields according to the magnetic saturation data in Table 2. An inconsistency emerges when comparing the magnetic saturation values and depolarization coefficients of the iron samples. Although the magnetization studies show a higher value for the nano-powders, the depolarization coefficient is nearly half that of the bulk Fe sample. Initially, potential errors in powder weight estimation due to the Teflon tape encapsulation method were considered. However, detailed analysis confirmed that such errors would be minor and insufficient to explain the observed discrepancy. This conclusion is further supported by calculations using the Halpern–Holstein equation (Eq. 3) when considering larger domain sizes in the bulk Fe than in the Fe-45 nm sample, enabled by the larger crystallite size and contributing to the corresponding depolarization differences.

These findings highlight a key trade-off: bulk materials, such as bulk Ni, yield higher depolarization signals, however, their larger particle sizes pose significant challenges for integration into porous fuel cell media. Conversely, Fe-45 nm nanoparticles, which have dimensions optimal for incorporation into GDL, exhibit lower depolarization signals but remain detectable at lower concentrations, substantially reducing the required sensor loading. To fully determine the optimal sensor material, however, temperature sensitivity must also be considered. Therefore, the following section examines the temperature dependence of each material, facilitating a comprehensive evaluation that carefully balances temperature sensitivity, signal detectability, and integration feasibility.

### Temperature dependence

The temperature sensitivity was analyzed through the magnetization and NDI measurements. As with other ferromagnetic materials, the magnetic saturation of iron and nickel decreases with temperature^46,47^. An effective way to visualize the thermal variation of this parameter is to use the *relative magnetic saturation variation* (k_Ms_), defined as follows:

11 $$\:{k}_{{M}_{s}}=\:\frac{{M}_{s}\:\left(T\right)}{{M}_{s}(30\:^\circ\:C)}$$

**Fig. 9:**
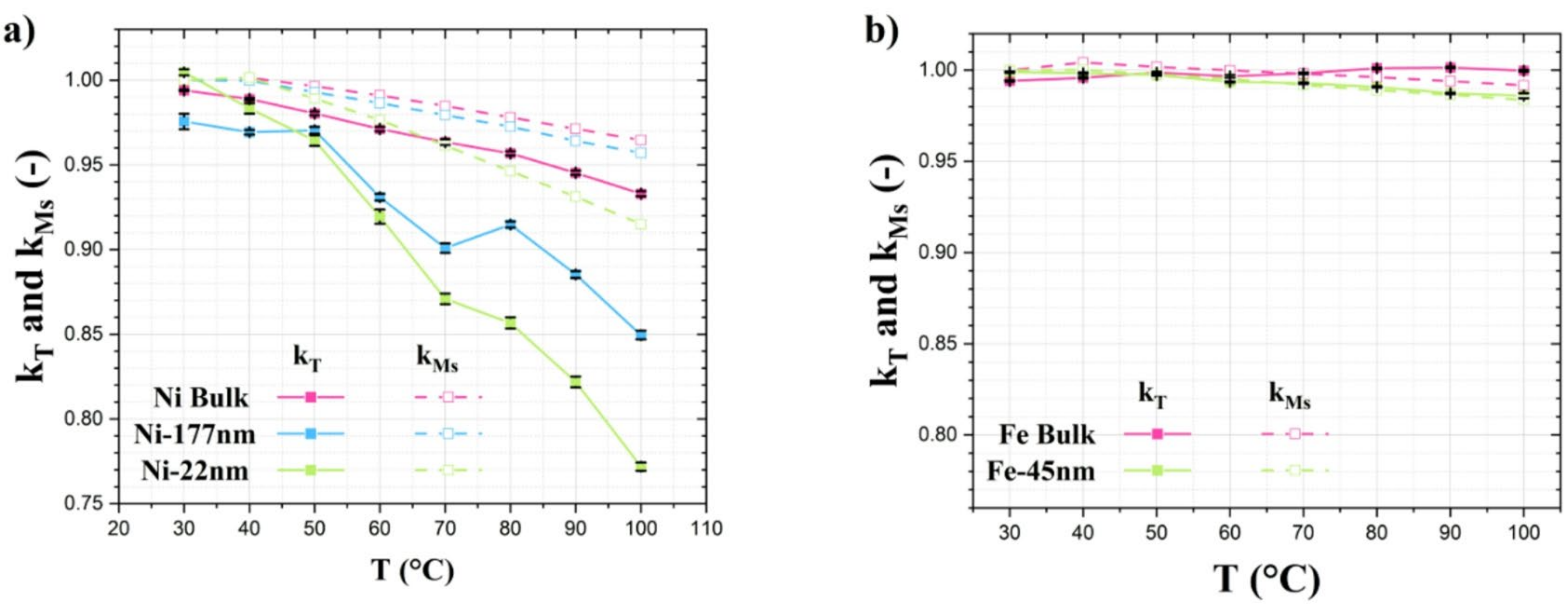
Relative magnetic saturation variation (dashed lines) compared with the relative depolarization variation (full line) for (a) nickel and (b) iron powders. All the samples are measured with a weight on weight (w/w) concentration of 10%, except for the Ni-22 nm sample where a concentration of 50% w/w is used.

Figure 9 depicts the observed temperature sensitivity comparing two distinct measurements: one involves neutron imaging, measuring the relative depolarization variation k_T_, while the other entails magnetization characterization, specifically quantifying the variation in thermal sensitivity k_Ms_ for the chosen samples.

In the k_Ms_, it is possible to observe a rather steep slope for nickel bulk in comparison to iron bulk. This behavior is unsurprising, given the difference in Curie temperatures (T_c_). As already evaluated by Dunlop et al. ^48^, the k_Ms_ tends to show an enhanced decreasing rate for temperature in T_c_ proximity. Another contribution of temperature sensitivity improvement is given by the dimensional effect, predominant in the case of nickel samples. In this case, a decrease in T_c_ value is observed below a particle size threshold, referred to as the critical radius size^49^. This behavior can be explained considering that T_c_ is dictated by the spin-spin exchange interaction. According to Ref. ^49^, the loss of magnetic ordering can be attributed to atomic bond breakage, a phenomenon particularly prominent in nanoparticles. The underlying cause can be attributed to the interplay between thermal energy and cohesive energy, with the latter being diminished due to the heightened energy instability observed in small nanoparticles. For the iron samples of this study, the Fe-45 nm thermal sensitivity remains almost unchanged with respect to the corresponding bulk material. This behavior indicates only a marginal decrease in T_c_, which, despite being insignificant, suggests that the particle size of the subsequent sample is in close proximity to, but not below, the critical radius described earlier.

The k_T_ also exhibit a notable temperature sensitivity for the nickel samples. In particular, a significant relative depolarization variation is observable in Ni-22 nm. This behavior confirms that the particle size affects the temperature sensitivity in this case. These results are consistent with the magnetization hysteresis and relative magnetic saturation variation, whose variations are more pronounced for nanoparticulate samples.

**Fig. 10:**
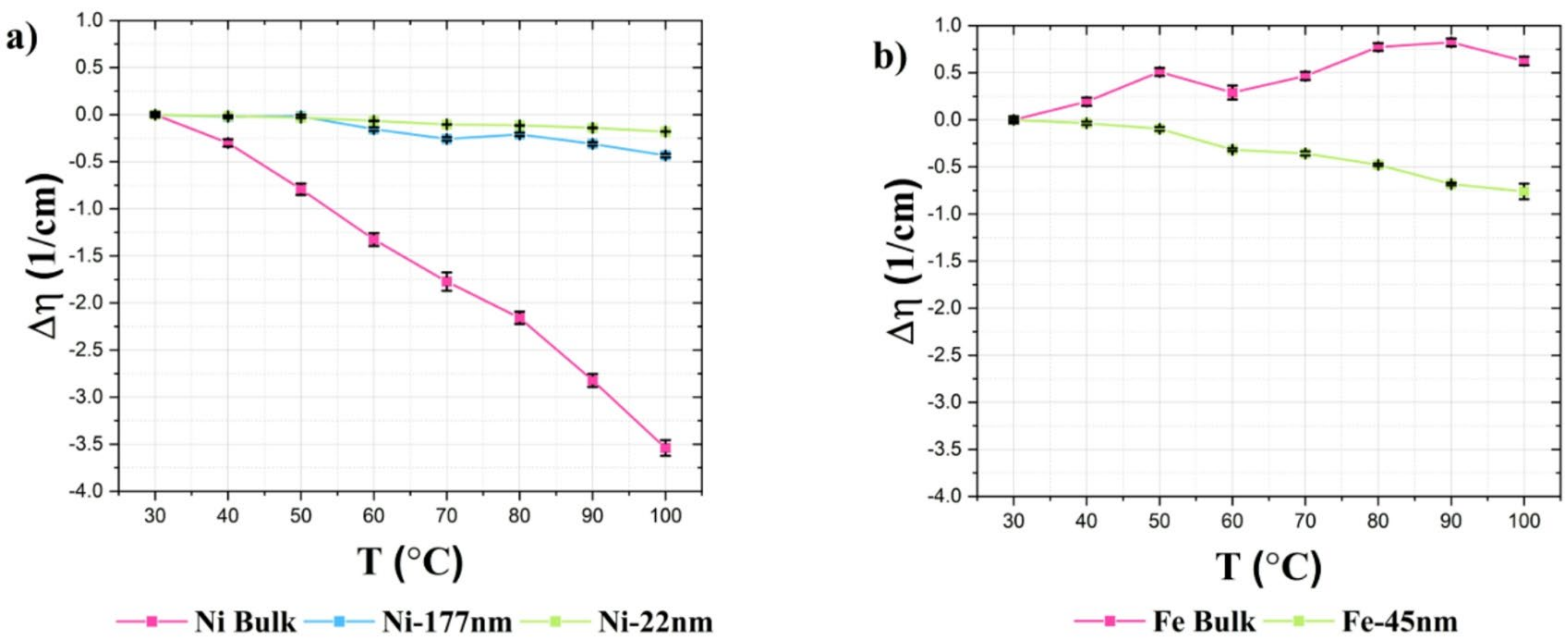
Absolute depolarization variation of (a) nickel and (b) iron powders across the temperature. All the samples are measured with a weight on weight (w/w) concentration of 10%, except for the Ni-22 nm sample where a concentration of 50% w/w is used.

While the relative depolarization variation holds significance for understanding the temperature sensitivity of the individual magnetic powders, it does not directly reflect imaging contrast, which is the measured signal within the context of depolarization imaging applications. To address this limitation, the absolute depolarization variation was introduced—a parameter that quantifies the extent of change in the depolarization coefficient respect its value at 30 °C. As discussed in the Data processing section, this parameter retains detection information, facilitating a more distinct differentiation of materials with stronger signals and furnishes a direct and precise indicator of sensitivity to temperature variations.

Initially, Fe-45 nm nanoparticles appeared promising due to their good depolarization signals and small particle size, ideal for integration into GDLs. However, considering the primary goal of accurate thermal sensing, temperature sensitivity becomes the most critical parameter. In this respect, Fe-45 nm nanoparticles do not demonstrate sufficient sensitivity. Conversely, bulk nickel clearly demonstrates superior temperature sensitivity, making it more suitable as a temperature sensor. The primary limitation of bulk nickel arises solely from its large particle size, which exceeds practical integration constraints. Hence, reducing nickel particle size to approximately 1 μm would preserve the excellent temperature-sensing properties of bulk nickel while ensuring practical feasibility in terms of integration in a cell, as further emphasized in the Conclusions section.

The highest values of *absolute depolarization variation* are obtained for bulk nickel (Fig. 10a), whereas its counterpart in iron (Fig. 10b) exhibits an unexplained anomalous behavior, which requires further analysis. Based on these results, Nickel Bulk would therefore constitute the optimum sensor material among the samples analyzed here. However, the aspect of integrating the material in a fuel cell GDL has to be considered as well. GDLs are made of fibers having a diameter of 10 μm and have pore sizes in the range of approximately 20 μm ^50^. Consequently, only materials of nanometric sizes up to a maximum of 1–2 μm can be considered for non-invasive integration into fuel cells.

Considering all experimental results, a clear performance hierarchy emerges among the tested powders. Bulk Ni provides the strongest depolarization signal and temperature sensitivity, however, its particle size (> 10 μm) exceeds the pore dimensions of typical GDLs. This size limitation motivated a shift to nanoparticulate candidates: Fe-45 nm readily fits the GDL porous and is detectable at low loadings, but its temperature response is inadequate for precise sensing, while Ni-22 nm displays excellent thermo-magnetic contrast only when used at concentrations ~ 7.5 times higher than Fe-45 nm—incompatible with non-invasive sensor deployment. Balancing signal strength, thermal sensitivity, and volumetric compatibility, nickel reduced to ~ 1 μm emerges as the optimal compromise, combining the superior sensing performance of bulk Ni with the physical dimensions required for integration.

### Correlation of NDI and magnetic properties

**Fig. 11:**
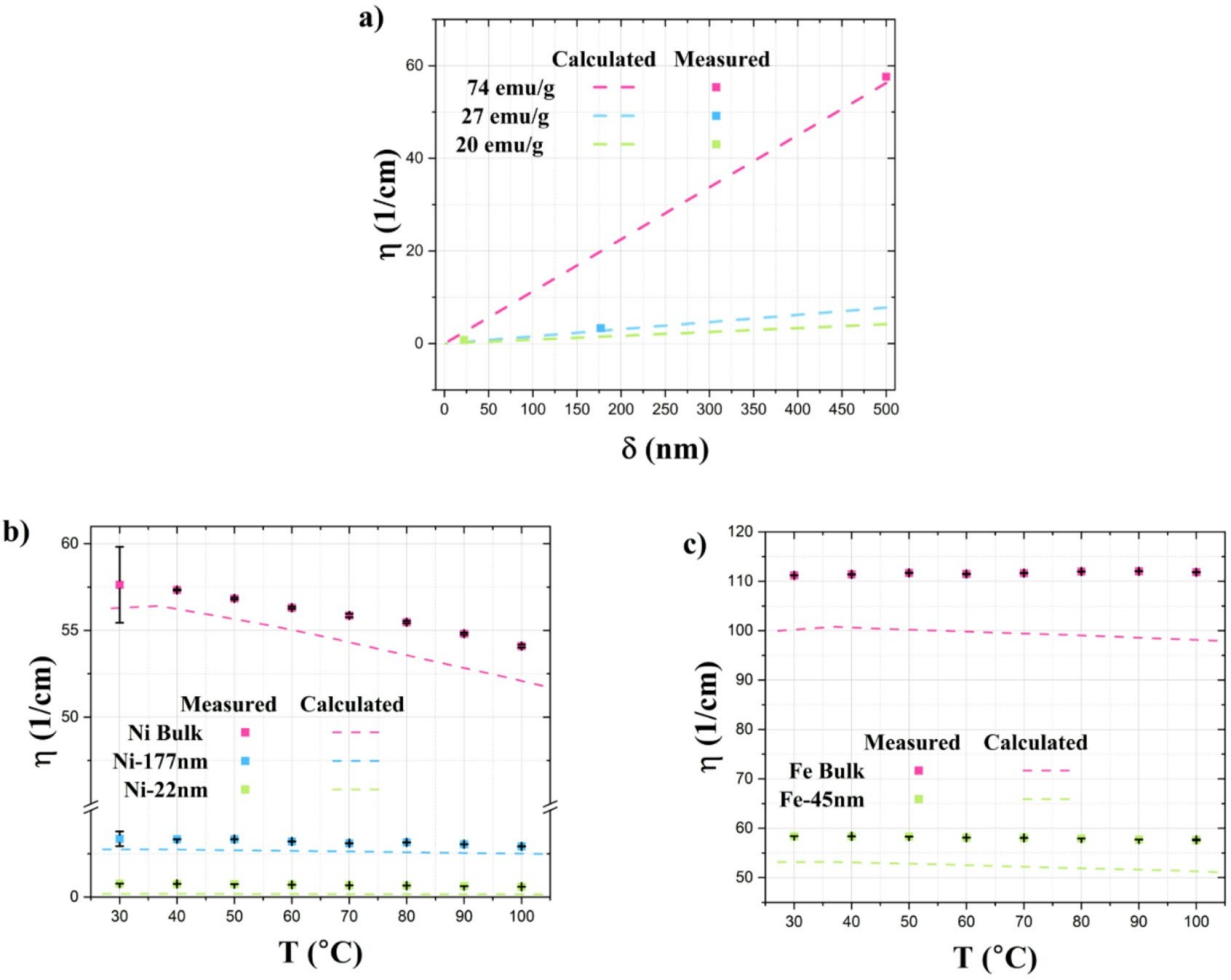
a) Neutron depolarization coefficient variation across domain size, calculated considering neutron with a wavelength λ = 5Å, in the range of domain size between 1 and 500 nm, for samples concentrated 10% w/w, in the case where the magnetic saturation is 74 emu/g (pink dashed line), and 27 emu/g (blue dashed line) and 20 emu/g (green dashed line), compared with the real data of nickel samples at 30 °C (blue dots). Comparison of calculated (dashed lines) and experimental (dots) depolarization coefficient of nickel (b) and iron (c) samples.

The central part of the discussion evaluated how the material properties can vary depending on several parameters. In the following section, the theoretical influence of particle size and magnetic saturation is estimated by applying the described statistical Eq. (3). In these calculations, the velocity of the neutrons utilized has been determined based on the weighted average of the neutron wavelengths, which corresponds to 5 Å. It has been assumed that the magnetic domains in these ferromagnetic materials are equal in size to their crystallites. This assumption is based on the understanding that the interaction between the magnetic moments and the crystal lattice is responsible for the formation and characteristics of the magnetic domains^51^. The effective thickness d_eff_, described in Eq. 4, incorporates volume fraction values and accounts for varying concentrations of these magnetic powders. Regarding the magnetic field, the saturation magnetization value was initially considered. However, a correction was necessary due to the partial reorientation induced by the guide fields (as shown in Fig. 3), which apply a magnetic field of 5.5 mT perpendicular to the neutron path. Consequently, by considering the magnetization hysteresis of each magnet powder, an interpolation of the magnetization value is performed with respect to the 5.5 mT value. The obtained value is then subtracted from the magnetic saturation value. Figure 11a shows how the particle size and magnetic saturation affect image contrast obtained through polarization contrast neutron imaging.

The distinction of the influence of these parameters in the calculated data is crucial when compared with the experimental data (Fig. 11b-c), where the sample’s crystallite size is assumed to be equal to their domain size, and the thermal dependency is calculated by applying the magnetic saturation at the defined temperature. The calculated values show good agreement with the experimental NDI data, confirming that it is possible to evaluate the Halpern and Holstein statistical equation as a helpful tool to estimate the neutron depolarization values by using the approximation of equality between crystallite and domain size, maintaining at the same time the information related to the temperature dependence. Based on these results, showing discrepancies of less than 10% between calculated and measured depolarization coefficients over the studied temperature range, we can conclude that the proposed model—assuming equivalence between crystallite size and domain size, as well as between magnetic saturation and the internal magnetic field within the domain—is valid and applicable for studying nickel and iron materials within our temperature-sensing investigations.

## Conclusions

By comparing the magnetic properties of ferromagnetic materials having different particle sizes and correlating these properties with the neutron depolarization coefficient of these materials, we have confirmed that smaller particles exhibit an increased temperature sensitivity. However, this advantage comes at the cost of a significantly reduced depolarization coefficient, a phenomenon explained by two combined effects. First, the saturation magnetization of a material is known to decrease for particle sizes in the nanometric range. Second, as established by the physics of neutron precession, a magnetic material with smaller magnetic domains will result in lower neutron depolarization. As a result, the choice of the ideal material for temperature sensing applications in fuel cells is a compromise between the particle size resulting in both a significant depolarization and a good temperature sensitivity. Combining these two factors, bulk nickel emerges as the most promising candidate, for which very good temperature sensing characteristics were obtained with a volume fraction as low as 2.4%. Further considerations arise for the integration in fuel cell materials, as the material particles must be sufficiently fine to be integrated in porous media. With the backing of the applied theory, we expect that Nickel particles having a size of 1 μm would keep the excellent sensing characteristics of the bulk Nickel, which being small enough for non-invasive integration into the porous structures of fuel cells. In the future, an in-situ implementation could be realized by embedding these particles using the fluorinated ethylene propylene (FEP) sintering process, where the binder melts during sintering.

## Data Availability

The datasets generated and/or analyzed during the current study are available from the corresponding author upon reasonable request.
